# Fuel oil generated from the cogon grass-derived Al–Si (*Imperata cylindrica* (L.) Beauv) catalysed pyrolysis of waste plastics

**DOI:** 10.1016/j.heliyon.2019.e02324

**Published:** 2019-08-17

**Authors:** Tapanee Sangpatch, Nuta Supakata, Vorapot Kanokkantapong, Bunjerd Jongsomjit

**Affiliations:** a Biotechnology Program, Faculty of Science, Chulalongkorn University, Bangkok, Thailand; b Department of Environmental Science, Faculty of Science, Chulalongkorn University, Bangkok, Thailand; c Department of Chemical Engineering, Faculty of Engineering, Chulalongkorn University, Bangkok, Thailand; d Research Program, Municipal Solid Waste and Hazardous Waste Management, Center of Excellence on Hazardous Substance Management (HSM), Bangkok, 10330, Thailand

**Keywords:** Materials science, Waste plastic, Pyroysis, Fuel oil, Cogon grass, Catalyst

## Abstract

This research investigated pyrolysis as a potential method to manage plastic waste in Sichang Island, Thailand. Pyrolysis was chosen to convert waste plastic into fuel oil using Al–Si catalysts derived from cogon grass. The study consisted of three stages. The first stage determined the composition of the waste plastics found in Sichang Island. High-density polyethylene (48%) comprised the highest proportion of the waste plastics, followed by low-density polyethylene (22%), polyethylene terephthalate (13%), polypropylene (10%), and polystyrene (7%). In the second stage, the Al–Si catalysts were prepared from cogon grass (*Imperata cylindrica* (L.) Beauv) by treating it with acid and calcination. The optimum conditions to extract silica from cogon grass through acid treatment were heating at 700 °C for 2 h, which yielded 97.7% of amorphous silica with a surface area of 172 m^2^/g and a pore volume of 0.43 cc/g. This amorphous silica was combined with an aluminum precursor to form Al–Si catalysts with 20–80 wt% of Al–Si. The results showed that the surface area of the catalyst increased with increasing aluminum content. The optimum ratio was 60 wt% of Al–Si with a surface area of 200 m^2^/g. In the final stage, the catalytic properties of the previously prepared Al–Si catalysts in the pyrolysis of waste plastics were evaluated. The catalyst enhanced the plastic cracking process and the oil yield while decreasing the reaction time. The optimum ratio of 60% Al–Si to 10% waste plastic provided the maximum oil yield of 93.11% and the minimum reaction time of 20 min. The results showed that catalytic cracking with 60% Al–Si contributed to a high quantity of oil yield, similar to using a commercial Al–Si catalyst. The results of this research will be applied as an alternative method of recycling plastic for sustainable waste management in Sichang Island.

## 1 Introduction

Municipal solid waste (MSW) is a critical problem in Thailand. The Pollution Control Department (PCD) reported that 27.4 million tons of MSW was produced in 2017, but only 8.52 million tons were recycled or reused. Although 11.70 million tons were properly disposed of, the remaining 7.18 million tons were improperly disposed of. During the last 15 years, the average per capita waste generation rate has increased from 0.62 kg/capita/day in 2002 to 1.13 kg/capita/day in 2017 [1]. According to the PCD report [2], the quantity of waste generated is expected to increase due to population and tourism growth [3]. Sichang Island, located in the Gulf of Thailand in Chonburi province, is one of Thailand's most popular tourist islands and has a total area of 25.61 square kilometers. The island has a convenient anchorage for shipping barges, and the rise in tourists and shipping activities has caused a tremendous increase in solid waste. According to the Sichang Municipality, the local government administration responsible for collecting and managing solid waste on the island, approximately 25 tons of solid waste are collected per day. Up to twenty percent of this waste (approximately 2–5 tons per day) is plastic [4]. Moreover, a large amount of plastic refuse has been found not only on the beach but also in the sea around Sichang Island [5, 6]. This waste takes hundreds of years to degrade [7]. Moreover, waste plastics can be broken down over decades into microplastics and can cycle into the human food chain [8]. Most litter worldwide, 60–80% of which is plastic [9], ultimately ends up in the sea; this effect has become a global concern [10, 11, 12].

Pyrolysis is considered an eco-friendly solution to reuse waste plastic, which forms the third largest proportion of MSW [13], as a fuel to conserve future energy [14, 15]. Pyrolysis entails thermal cracking of long-chain hydrocarbons into short-chain hydrocarbons at temperatures from 300 to 600 °C to obtain liquid oil as an energy product and char and gases as value-added products [16, 17]. The pyrolysis of municipal plastic waste into liquid oil as an energy source has recently received greater attention [18, 19, 20]. There are four principal steps in pyrolysis: initiation, transfer, decomposition, and termination [21].

The introduction of catalysts into the pyrolysis process improves the rate of the cracking reactions and reduces the process temperature and retention time [22, 23]. The crucial properties of catalysts, including surface area, acidity, pore size, and pore volume, are the main factors that affect pyrolysis [24]. The catalysts FCC, silica-alumina, MCM-41, and zeolite have been used in plastic waste pyrolysis. FCC, a microporous catalyst, can restrict the entrance of hydrocarbons into the catalyst and enhance the quality and quantity of liquid oil yield, whereas zeolites such as ZSM-5 increase the gases' yield because of their mesoporous structure [25]. Silica–alumina, one of the most widely used catalysts, is an amorphous acid catalyst consisting of Bronsted acid sites with ionizable hydrogen atoms and Lewis acid sites as electron acceptors that require low acidity and a high processing temperature. These catalysts can all enhance liquid oil production [25]. Usually, biomass is used as a pure silica source due to its low commercial value; it is prepared through acid leaching and calcination [26, 27]. Cogon grass (*Imperata cylindrical* (L.) Beauv.), one of the most invasive weeds in the world, is commonly found in Thailand and Southeast Asia [27, 28]. It has been recommended as a source of silica [27, 29]. Bunmai et al. [27] showed that treating cogon grass’ glassy blade and trunk with hydrochloric acid (HCl) and calcination at 500 °C for 4 h can produce high-purity silica, while Thongrut and Kueansombat [30] found that calcination of cogon grass at 600 °C obtained the highest yield of silica (65.55%).

In this research cogon grass was therefore used to produce Al–Si catalysts in pyrolysis to convert waste plastic into fuel oil. The study comprised three stages. The first stage characterized the composition of the waste plastics in Sichang Island. The second stage prepared the Al–Si catalysts from cogon grass with acid treatment and calcination. The final stage evaluated the Al–Si catalysts prepared from cogon grass in the pyrolysis of waste plastics to obtain fuel oil. The findings of this research should help facilitate an alternative option for managing waste plastic in Sichang Island.

## 2 Materials and methods

### 2.1 Materials

#### 2.1.1 Waste plastic

Waste plastics were obtained from Sichang Island in Chonburi province, Thailand. The American Society for Testing and Materials (ASTM) D5231-92 standard test method was applied for analyzing the composition of municipal waste plastics [31].

#### 2.1.2 Cogon grass

Two kg of cogon grass were collected from Surin province in northeastern Thailand. The grass was cut into 15 cm lengths, washed, and dried in the oven at a temperature of 105 °C for 24 h. The chemical composition was analyzed using an X-ray fluorescence spectrometer (S8 Tiger, Bruker).

### 2.2 Methods

#### 2.2.1 Silica extraction

To extract silica, the cogon grass was refluxed in an HCl solution with a concentration of 0.1M (1000 g of cogon grass/100 ml of HCl) at a temperature of 90 °C for 10 h. Then, the cogon grass was calcined at temperatures of 600 °C and 700 °C for 2 h and 4 h, respectively. The chemical composition of the extracted silica from cogon grass was analyzed using an X-ray fluorescence spectrometer (S8 Tiger, Bruker), and the patterns of the silica extracted from cogon grass were analyzed by X-ray diffraction (D8 Discover). A microstructural characterization of the materials was identified by using a scanning electron microscope (Jeol JSM-6480LV). The Brunauer-Emmett-Teller (BET) surface area and pore volume of the extracted silica were analyzed using a BET surface area analyzer.

#### 2.2.2 Silica-alumina catalyst synthesis

Four atomic ratios (wt%) of Al–Si (20:80, 40:60, 60:40, and 80:20) were prepared. The nomenclature of the catalysts is as follows: Al–Si/20–80, Al–Si/40–60, Al–Si/60–40, and Al–Si/80–20. These Si precursors were mixed for 60 min with aluminum isopropoxide and isopropanol; extracted silica from cogon grass (see section 2.2.1) was added under stirring for 60 min and aged at ambient temperature by 24 h. The final mixture was treated at 110 °C for 24 h and calcined at 650 °C for 2 h. The chemical composition of the synthesized Al–Si catalysts was analyzed using an X-ray fluorescence spectrometer (S8 Tiger, Bruker), and the patterns of the extracted silica from cogon grass were analyzed by X-ray diffraction (D8 Discover). A microstructural characterization of the materials was identified using a scanning electron microscope (Jeol JSM-6480LV). The BET surface area and pore volume were analyzed using a BET surface area analyzer.

#### 2.2.3 Pyrolysis process

The collected feedstock samples of waste plastics from Sichang Island were mainly composed of PET, HDPE, LDPE, PP, and PS plastic types. The feedstock was washed, dried, and cut into small sizes (0.5 cm^2^) to obtain a homogenous mixture for the reactor.

**Fig. 1:**
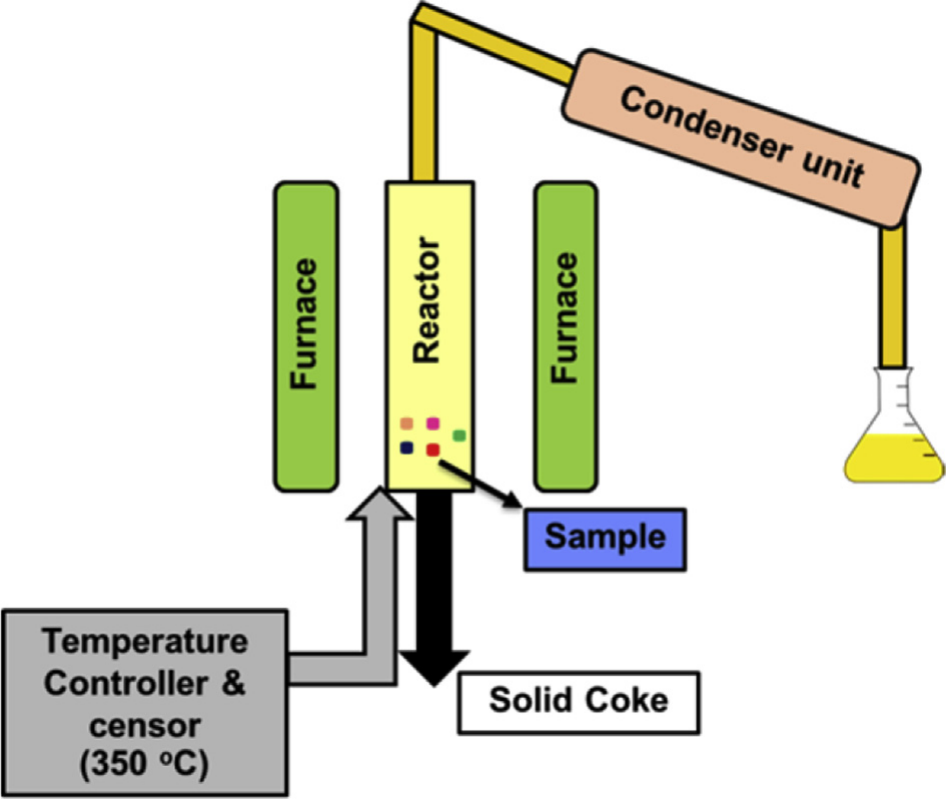
Small laboratory-scale pyrolysis reactor.

The prepared feedstock sample (15 g) was put into the heating reactor for pyrolysis. In this process, plastic feedstock was converted into organic monomers in the reactor, and these monomers were later condensed into liquid oil in the condenser as shown in Fig. 1. Kache [32] studied using catalysts in cracking high-density polyethylene and polypropylene at 350 °C and found that increasing the temperature higher than 400 °C caused the glass-type reactor to melt and deform. In this study the feedstock was therefore heated at 350 °C.

At the end of each experiment, the obtained yield was calculated by using Eqs. (1), (2), and (3).

(1) $$\text{Yield}\;\text{of}\;\text{liquid}\;\text{oil}\;\left(\text{\%}\right)\;=\frac{weight\;of\;liquid\;oil}{weight\;of\;feedstock}\;\;\text{× ​}\;100$$

(2) $$\text{Yield}\;\text{of}\;\text{char}\;\left(\text{\%}\right)\;=\frac{weight\;of\;carbon\;black}{weight\;of\;feedstock}\text{× ​}\;100$$

(3) Yieldofgases(%)=100−(%yieldofliquidoil+%yieldofcarbonblack)

A small laboratory-scale pyrolysis reactor (a glass-type reactor under atmospheric pressure in batch operation) was commissioned and used to perform the catalytic pyrolysis of waste plastics by varying the four ratios of catalysts mentioned in section 2.2.2. Experimentally, 10 wt% of each catalyst was mixed with the feedstock plastic to determine the effect of their catalytic properties on the yield and calorific value of the liquid oil product. In fact, the 10wt% of Al–Si catalyst exhibits the moderate acid sites to accelerate the reaction. If the amounts of Al increase, the acidity will increase as well. Too high acidity apparently results in higher coke formation (carbon deposition on the surface of catalyst) leading to rapid deactivation of catalysts. Thus, moderate acidity is necessary. The condensed liquid oil was collected from the oil collection assembly and calculated for yield (eq. 1) and calorific value (Bomb Calorimeter Parr 6200). The Al–Si catalyst exhibiting the highest yield of liquid oil was chosen to further examine the optimal ratio of Al–Si catalyst to feedstock plastic (wt%) by comparing 20:80 and 30:70 ratios to find which had the highest liquid oil yield and calorific value.

## 3 Results and discussion

### 3.1 Waste plastic composition

As shown in Table 1, HDPE from plastic bags, milk bottles, and food packaging boxes and containers is the major constituent of the waste plastic generated on Sichang Island, representing 48% of the total waste plastic generated, while LDPE from plastic packaging bags and PET mostly from water and drink bottles constituted 22% and 13%, respectively.

**Table 1:** Composition of waste plastic generated on Sichang Island.

Waste plastic type	%
High density polyethylene: HDPE	48
Low density polyethylene: LDPE	23
Polyethylene terephthalate: PET	13
Polypropylene: PP	8
Polystyrene: PS	7
Polyvinyl chloride: PVC	1

PS, PE (HDPE and LDPE), and PP are suitable for pyrolysis [25], while PVC is not suitable because it produces hazardous chlorine gas, must be dechlorinated at a temperature between 250-320 °C, and affects the catalytic activity in pyrolysis because of the appearance of chlorine and the deposition of coke [33, 34]. PS produces high-quality fuel because it requires lower temperatures and produces less viscous oil than PE and PP, which require high temperatures because of their complex branched chain structure [35, 36, 37]. In this research, mixed plastic waste (excluding PVC) with a ratio of 48 HDPE:22 LDPE:13 PET:10 PP:7 PS was selected (similar to the waste plastic composition of Sichang Island).

### 3.2 Composition and phases of silica from calcined cogon grass

Silica was the major component of all the calcined cogon grass samples (Table 2). The results showed that the silica of cogon grass after it was prepared ranged from 97.30 to 97.70 wt%, which is higher than that of untreated cogon grass (1.5 wt%) due to the removal of impurities by hydrolysis activity [27]. A small amount of other inorganic oxides, including P_2_O_5_, CaO, Fe_2_O_3_, SO_3_, Al_2_O_3_, ZnO, MgO, and K_2_O was observed.

**Table 2:** Chemical composition, BET surface area, and micropore volume of calcined cogon grass.

Composition (% wt.)	Calcined at 600 °C		Calcined at 700 °C	
	2 h	4 h	2 h	4 h
SiO_2_	97.30	97.70	97.70	97.50
P_2_O_5_	0.91	0.90	0.70	0.72
CaO	0.43	0.36	0.47	0.53
Fe_2_O_3_	0.33	0.25	0.23	0.26
SO_3_	0.23	0.14	0.17	0.26
Al_2_O_3_	0.23	0.19	0.13	0.19
ZnO	0.20	0.16	0.27	0.15
MgO	0.14	0.12	0.14	0.15
K_2_O	0.16	0.14	0.10	0.14
Others	0.07	0.04	0.09	0.10
BET surface area (m^2^g^−1^)	130	108	172	90
Total pore volume (cm^3^g^−1^)	0.37	0.31	0.45	0.34

Cogon grass after leaching with 0.1 M HCl and calcination at 600 °C and at 700 °C for 2h and 4 h, respectively, had a broad peak at 22^°^ indicating that amorphous silica is the main phase; the XRD pattern of silica extracted by acid treatment and calcination at 700 °C for 2 h was similar to that in the literature [27].

As shown in Table 2, cogon grass after leaching with 0.1 M HCl and calcination at 700 °C for 2h obtained the highest BET surface area and a total pore volume of 172 m^2^g^-1^ and 0.45 cm^3^g^-1^. In general, increased temperature and time will result in better pore size distribution and increased surface area at some extent values. However, when too much high temperature and time are used, it will lead to sintering of pore. As the result, the surface area remarkably decreases, while the pore size increases. Thus, both temperature and time must be optimized to obtain suitable pore size and surface area. In general, increased temperature and time will result in better pore size distribution and increased surface area at some extent values. However, when too much high temperature and time are used, it will lead to sintering of pore. As the result, the surface area remarkably decreases, while the pore size increases. Thus, both temperature and time must be optimized to obtain suitable pore size and surface area.

These results were consistent with the XRD analysis, which showed that the structure of the amorphous silica treated at 700 °C for 2 h had the highest surface area and porosity and was suitable to use as a receptor in catalyst production.

### 3.3 Characterization of the synthetic Al–Si catalyst from cogon grass

The XRF analysis of the synthetic Al–Si catalyst from cogon grass showed that Al_2_O_3_ increased as the percentage of alumina increased, while SiO_2_ decreased (Table 3). The synthetic Al–Si catalyst from cogon grass with 60% alumina obtained the highest BET surface area and a total pore volume of 200 m^2^g^-1^ and 0.72 cm^3^g^-1^.

**Table 3:** Chemical composition, BET surface area, and micro-pore volume of the synthetic Al–Si catalyst from cogon grass.

Composition (% wt.)	Al–Si/20-80	Al–Si/40-60	Al–Si/60-40	Al–Si/80-20
SiO_2_	73.20	60.90	37.60	21.50
Al_2_O_3_	24.30	37.20	61.10	77.90
CaO	0.71	0.53	0.41	0.15
SO_3_	0.53	0.47	0.29	0.23
P_2_O_5_	0.38	0.26	0.19	0.00
Na_2_O	0.36	0.31	0.19	0.00
MgO	0.16	0.00	0.00	0.00
Fe_2_O_3_	0.15	0.12	777 ppm	684 ppm
K_2_O	0.11	760 ppm	529 ppm	300 ppm
Others	0.10	0.21	0.22	0.22
BET surface area (m^2^g^−1^)	102	143	200	163
Total pore volume (cm^3^g^−1^)	0.30	0.47	0.72	0.58

**Fig. 2:**
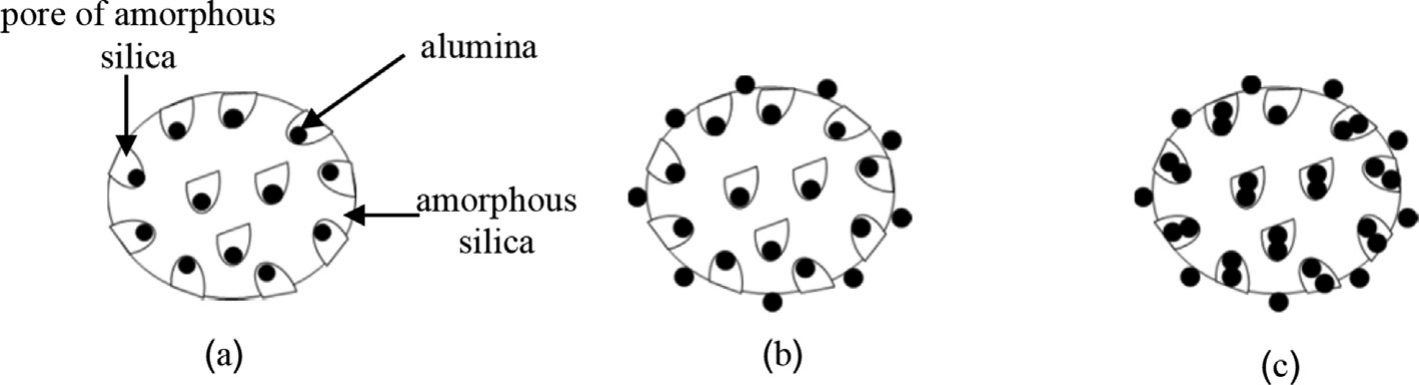
Surface area of amorphous silica: (a) Al–Si/20–80 and Al–Si/40–60; (b) Al–Si/60–40; (c) Al–Si/80-20.

The results of the BET surface area in Table 3 show that the surface area of the Al–Si catalysts decreased when loading amorphous silica with 20 and 40 wt% of alumina because the alumina was precipitated and deposited into the pores of the amorphous silica (Fig. 2a). However, when the ratio of alumina increased to 60 wt%, the surface area of the amorphous silica was increased higher than that of the pretreated amorphous silica with alumina, due to the diffusion of alumina into the amorphous silica surface, which increased the surface area of the amorphous silica (Fig. 2b). When the concentration of alumina increased to 80 wt%, the surface area of the amorphous silica decreased, due to the high amount of alumina diffused, precipitated, and deposited in the pores and outer surface, reducing the surface area of the amorphous silica (Fig. 2c). According to Miandad et al. [25], the initial cracking takes place at the outer surface of the catalyst, and later cracking takes place when a molecule enters into the catalyst pore; thus, the silica-alumina catalysts from cogon grass with the ratio of 60 wt% silica obtained the highest surface area and pore volume for cracking.

**Fig. 3:**
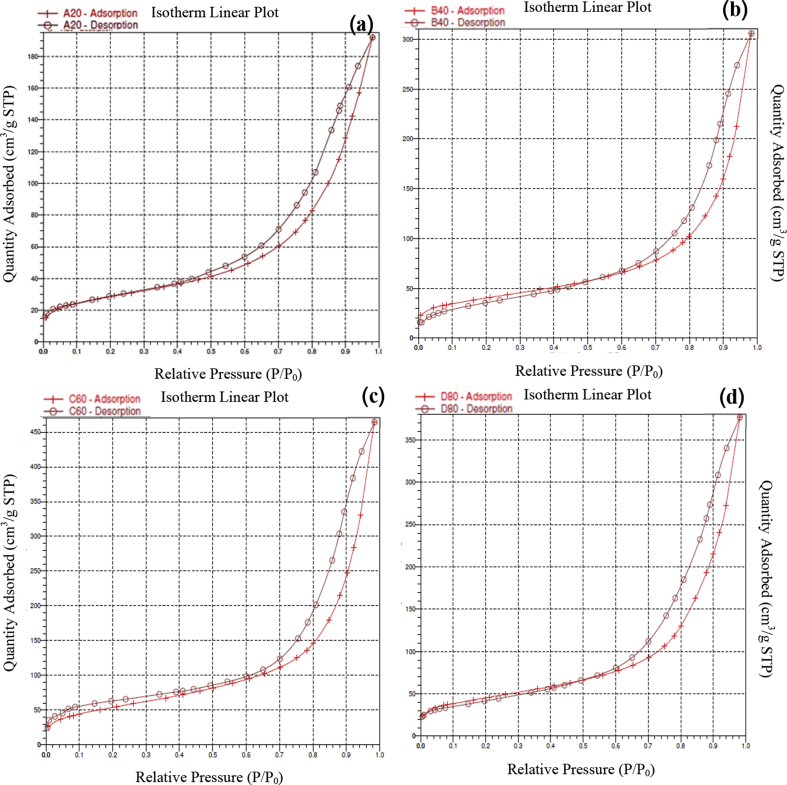
Adsorption-desorption isotherm of the Al–Si catalyst from cogon grass: (a) Al–Si/20–80 (b) Al–Si/40–60; (c) Al–Si/60–40; (d) Al–Si/80-20.

In addition, the results of isotherm analysis (Fig. 3) show that the adsorption-desorption isotherm of the silica-alumina catalyst from cogon grass had a medium-sized porous type IV (mesoporous) with a high surface area suitable to use as a catalyst.

**Fig. 4:**
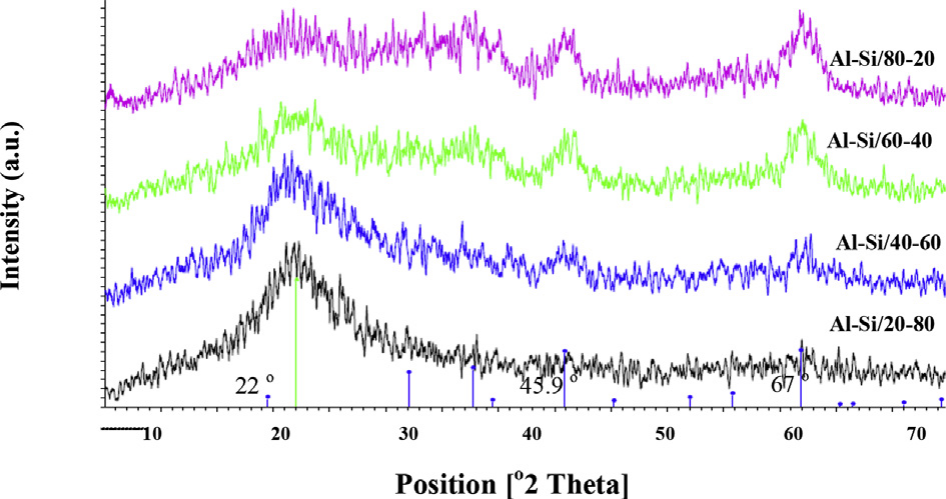
XRD patterns of the synthetic Al–Si catalyst from cogon grass.

**Fig. 5:**
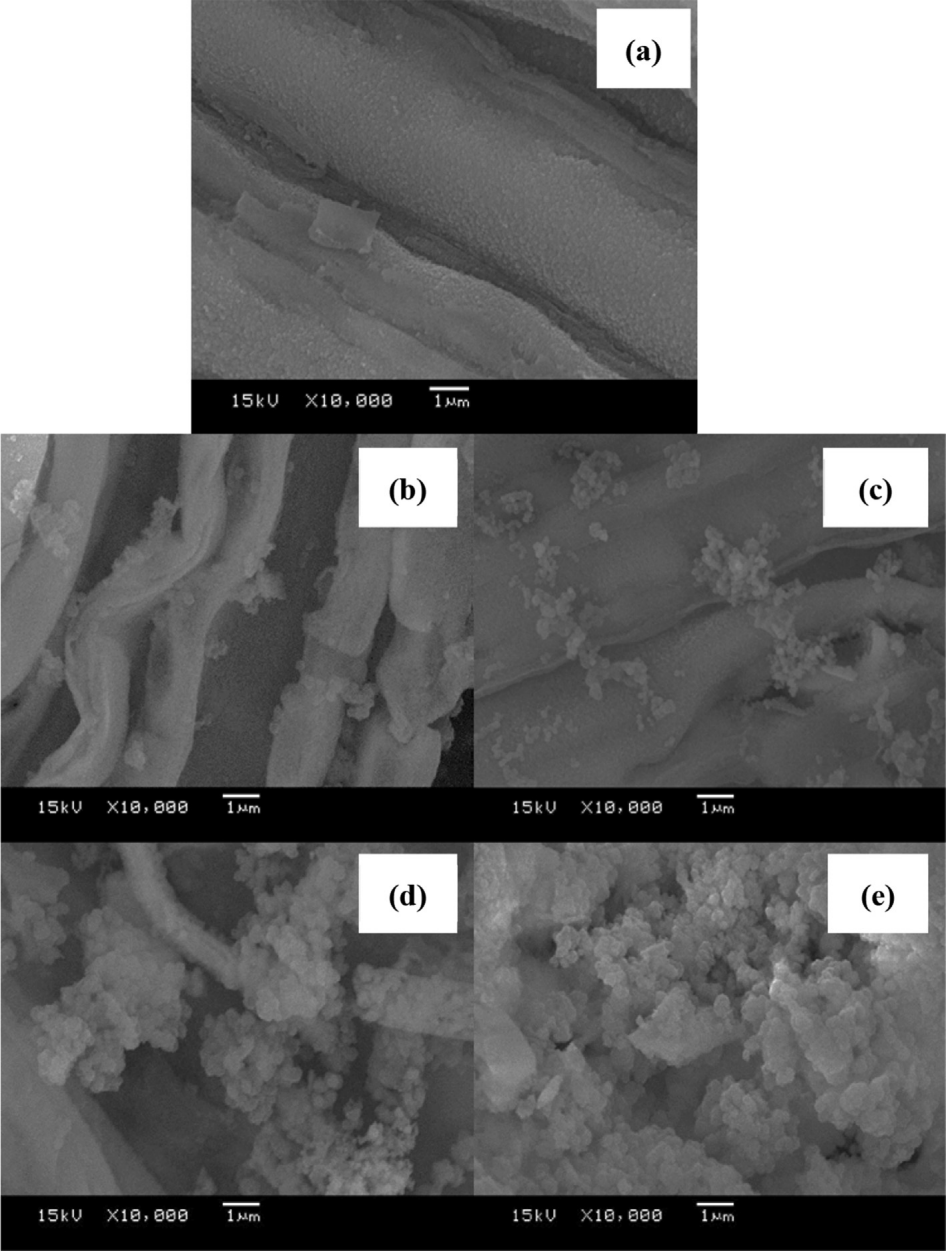
SEM images of the synthetic Al–Si catalyst from cogon grass: (a) w/t catalyst (b) Al–Si/20–80 (c) Al–Si/40–60; (d) Al–Si/60–40; (e) Al–Si/80-20.

Fig. 4. Shown the structure of synthetic catalysts at Al–Si/20–80, Al–Si/40–60, Al–Si/60–40 and Al–Si/80–20, the results found that silica obtained from calcination cogon grass at 700 °C for 2 h occurred broad peak at 22.0^°^ was the amorphous silica. This figure shows that when aluminate was added at 20, 40, 60 and 80 wt%, significant peaks occurred at 45.9 °C and 67 °C, which is a characteristic of Al_2_O_3_ (Gamma-Alumina). The peaks were more intense when the alumina content increased from a ratio of 40%, supported by the SEM images in Fig. 5, in which the presence of amorphous alumina increased as the percentage of alumina rose.

Fig. 5 evidenced the XRD results in Fig. 4 that the higher absorption of alumina, the rougher surface of silica. This roughness can increase the surface area of silica. This study found that the optimal ratio of the synthetic Al–Si catalyst from cogon grass obtained the highest surface area was Al–Si/60–40 as describes in Fig. 2.

### 3.4 Effect of the Al–Si catalyst from cogon grass on liquid oil yield from waste plastic pyrolysis

To investigate the optimum conditions for thermal decomposition under controlled conditions, 15 g of mixed waste plastic feedstock with the ratio of 48 wt% HDPE:22 wt% LDPE:13 wt% PET:10 wt%PP:7 wt% PS was used in the pyrolysis reactor. The reactor was heated from room temperature to 350 °C at a rate of 5 °C per min; while the catalyst was absent the feedstock could be examined to determine the optimum conditions for thermal decomposition under controlled conditions. The results showed that pyrolysis at the temperature of 350 °C for 30 min obtained a maximum conversion of feedstock into liquid oil (84.66%), char (14.93%), and gases (0.41%).

**Fig. 6:**
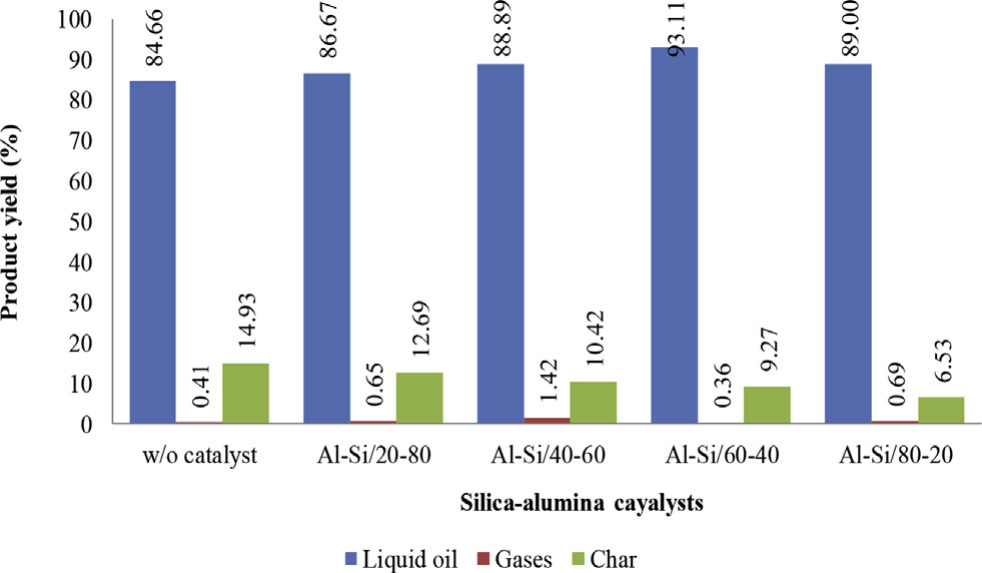
Percentage of oil yield using the catalyst from cogon grass in the waste plastic cracking process.

Afterwards, 10% by weight of the Al–Si catalyst from cogon grass with concentrations of 20, 40, 60, and 80 wt% alumina was used in the pyrolysis of waste plastic. As presented in Fig. 6, the results show that the pyrolysis of waste plastic using the Al–Si catalyst from cogon grass with a concentration of 60 wt% alumina obtained the highest liquid oil yield (93.11%) because it had the highest BET surface area and pore volume, which led to more cracking [25].

To compare oil yield from the catalytic pyrolysis of plastic waste with synthetic Al–Si catalysts from cogon grass and other catalysts including Y-Zeolite, B-Zeolite, HZSM-5 [38-41] shown in Table 4, it was found that the Al–Si/60–40 catalyst from cogon grass produced oil yield (93.11%) as the commercial Al– Si catalysts (93.22%) [40] due to the catalyst obtained from cogon grass had total surface area (200 m^2^/g) as commercial Al– Si catalysts (235 m^2^/g). In addition, synthetic Al–Si catalysts from cogon grass used the temperature in the pyrolysis process lower than the commercial Al– Si catalysts. From these results, it can be concluded that the synthetic Al–Si catalysts from cogon grass has the catalytic ability to produce fuel oil from plastic waste pyrolysis as the commercial catalysts do.

**Table 4:** The comparison of oil yield from the catalytic pyrolysis of plastic waste with synthetic Al–Si catalysts from cogon grass and other catalysts.

Catalyst	Si–Al ratio	Surface area (m^2^/g)	Plastic type	Temperature (^°^C)	Oil Yield (%)	Gas Yield (%)	Solid Residual (%)	Ref.
Al–Si/20-80	20	102	HDPE, LDPE, PP, PS, PET	350	86.67	0.65	12.46	This study
Al–Si/40-60	40	143	HDPE, LDPE, PP, PS, PET	350	88.89	1.42	10.67	This study
Al–Si/60-40	40	200	HDPE, LDPE, PP, PS, PET	350	93.11	0.36	9.27	This study
Al–Si/80-20	80	163	HDPE, LDPE, PP, PS, PET	350	89.00	0.69	6.53	This study
Y-Zeolite	80	-	HDPE	500	55.00	38.00	7.00	[38]
B-Zeolite	17.1	349	HDPE, LDPE, PP, PS, PET, PVC, Other	500	46.8	27.9	25.3	[39]
HZSM-5	12.6	367	HDPE, LDPE, PP, PS, PET, PVC, Other	500	43.8	34.6	21.6	[39]
Commercial Al–Si	13.4	235	LLDPE	450	93.20	2.2	4.6	[40]
Commercial Al–Si	35.13	29	PP	500	91	8	1	[41]

The heating value is an important property of liquid oil used as an alternative source of energy [25, 42]. In this study the heating value of liquid oil produced by waste plastic pyrolysis with or without using the catalyst from cogon grass was in the range of 45.02–45.54 MJ/kg (Table 5). Due to the high heating value of liquid oil from waste plastic, Saptoadi and Pratama [43] showed that it has the capability to substitute for kerosene oil and could possibly be used as an alternative fuel to conventional diesel [42].

**Table 5:** Heating value of liquid oil from waste plastic pyrolysis using the silica-alumina catalyst from cogon grass.

Heating value (MJ/kg)				
w/t catalyst	Al–Si/20-80	Al–Si/40-60	Al–Si/60-40	Al–Si/80-20
45.02	45.54	45.32	45.22	45.15

To compare heating value of liquid oil from the catalytic pyrolysis of plastic waste with synthetic Al–Si catalysts from cogon grass and other researches [38, 42, 44] shown in Table 6, it was showed that the synthetic Al–Si catalysts from cogon grass used lower temperature (350 °C) in pyrolysis process than others (450–500 °C) to gain comparable heating value.

**Table 6:** The comparison of heating value of liquid oil from the catalytic pyrolysis of plastic waste with synthetic Al–Si catalysts from cogon grass and others.

Catalyst	Plastic Type	Temperature (^°^C)	Heating Value (MJ/kg)	Ref.
w/t catalyst	HDPE, LDPE, PP, PS, PET	350	45.02	This study
Al–Si/20-80	HDPE, LDPE, PP, PS, PET	350	45.54	This study
Al–Si/40-60	HDPE, LDPE, PP, PS, PET	350	45.32	This study
Al–Si/60-40	HDPE, LDPE, PP, PS, PET	350	45.22	This study
Al–Si/80-20	HDPE, LDPE, PP, PS, PET	350	45.6	This study
MgCO_3_	HDPE	450	45.15	[44]
w/t catalyst	PE, PS, PET, PP	450	41.8	[42]
Y-Zeolite	HDPE	500	42.82	[38]

However, waste plastic pyrolysis produces a low-quality liquid oil with impurities and a high percentage of aromatic hydrocarbons [42] from the metal and acid sites in the catalyst. The presence of impurities, including sulfur, chlorine, solid residue, moisture, and acids from waste plastic pyrolysis reduces the quality of the liquid oil and limits its commercial applications [42]. Therefore, post-treatment, such as filtration or chemical treatment by blending with other fuels, distilling, and refining, is required to remove impurities and to improve its quality [42]. In order to manage waste plastic, Wang et al. [45] proved by Life Cycle Assessment (LCA) that pyrolysis is a good choice for waste plastic management.

## 4 Conclusions

This study investigated the role of Al–Si catalysts from cogon grass in pyrolysis used to produce fuel oil from waste plastic. The study hypothesized that Al–Si catalysts derived from cogon grass could be used in pyrolysis to convert waste plastic into fuel oil as an alternative plastic waste management method in Sichang Island. This study consisted of three stages: composition analysis of waste plastic in Sichang Island, preparation of Al–Si catalysts from cogon grass by acid treatment and calcination, and pyrolysis of waste plastic using Al–Si catalysts from cogon grass. The results can be summarized as follows:

− HDPE from plastic bags, milk bottles, and food packaging boxes and containers is the major proportion of the waste plastic generated on Sichang Island at 48%, followed by LDPE (22%) from plastic packaging bags, PET (13%) from water bottles, PP (8%), PS (7%), and PVC (1%) respectively.
− Cogon grass after leaching with 0.1 M HCl and calcination at 700 °C for 2h obtained the highest surface area and porosity and is suitable to use as a receptor in catalyst production.
− The Al–Si catalysts from cogon grass with the ratio of 60 wt% silica obtained the highest surface area and pore volume due to the diffusion of alumina into the amorphous silica surface, increasing the surface area of the amorphous silica.
− The pyrolysis of waste plastic using the Al–Si catalyst from cogon grass with the concentration of 60 wt% alumina obtained the highest liquid oil yield (93.11%).

To apply this research as an alternative plastic waste management method on Sichang Island, a pilot experiment, an economic feasibility study, and a life cycle assessment should be investigated.

## Declarations

### Author contribution statement

Nuta Supakata: Conceived and designed the experiments; Analyzed and interpreted the data; Contributed reagents, materials, analysis tools or data; Wrote the paper.

Tapanee Sangpatch: Conceived and designed the experiments; Performed the experiments; Analyzed and interpreted the data.

Bunjerd Jongsomjit: Analyzed and interpreted the data; Contributed reagents, materials, analysis tools or data.

Vorapot Kanokkantapong: Analyzed and interpreted the data.

### Funding statement

This work was supported by the Office of Higher Education Commission and the S&T Postgraduate Education and Research Development Office under the Research Program (grant HSM-PJ-CT-17-02).

### Competing interest statement

The authors declare no conflict of interest.

### Additional information

No additional information is available for this paper.
